# Effect of Submerged Entry Nozzle Shape on Slag Entrainment Behavior in a Wide-Slab Continuous Casting Mold

**DOI:** 10.3390/ma19030460

**Published:** 2026-01-23

**Authors:** Guangzhen Zheng, Lei Ren, Jichun Yang

**Affiliations:** School of Rare Earth Industry, Inner Mongolia University of Science and Technology (IMUST), Baotou 014010, China; zhengguangzhen1206@163.com (G.Z.); yangjichun1963@163.com (J.Y.)

**Keywords:** wide slab, meniscus fluctuation, slag entrainment, nozzle shape, water model

## Abstract

Slag entrainment within the mold is a significant cause of surface defects in continuously cast slabs. As a key component for controlling molten steel flow, the structure of the submerged entry nozzle directly influences the flow field characteristics and slag entrainment behavior within the mold. This paper employs a 1:4-scale water–oil physical model combined with numerical simulation to investigate the effects of elliptical and circular submerged entry nozzles on slag entrainment behavior in a wide slab mold under different casting speeds and immersion depths. High-speed cameras were used to visualize meniscus fluctuations and oil droplet entrainment processes. An alternating control variable method was employed to quantitatively delineate a slag-free “safe zone” and a “slag entrainment zone” where oil droplets fall, determining the critical casting speed and critical immersion depth under different operating conditions. The results show that, given the nozzle immersion depth and slag viscosity, the maximum permissible casting speed range without slag entrainment can be obtained, providing a reference for industrial production parameter control. The root mean square (RMS) of surface fluctuations was introduced to characterize the activity of the meniscus flow. It was found that the RMS value decreases with increasing nozzle immersion depth and increases with increasing casting speed, showing a good correlation with the frequency of slag entrainment. Numerical simulation results show that compared with elliptical nozzles, circular nozzles form a more symmetrical flow field structure in the upper recirculation zone, with a left–right vortex center deviation of less than 5%, resulting in higher flow stability near the meniscus and thus reducing the risk of slag entrainment.

## 1. Introduction

Slab quality has always been a core issue in continuous casting. Developing slabs with fewer defects and higher quality is an important goal for the continued development of continuous casting technology. As the “heart” of the continuous casting machine, the mold is the final link in controlling the cleanliness of molten steel and is also the main source of most internal and surface defects in the slab [1,2,3,4,5]. The flow state of molten steel in the mold has a decisive influence on slab quality. An unstable flow field can easily lead to increased fluctuations of the meniscus level, causing the protective slag to detach from the steel–slag interface and be entrained into the mold. The entrained protective slag can be captured by the solidified slab shell, forming slag-bearing defects that seriously affect the surface quality of continuously cast slabs.

In actual casting, due to the high-temperature and non-transparent environment inside the mold, the transient flow behavior of molten steel is difficult to observe directly. Therefore, researchers have generally employed physical simulation [6,7,8,9,10] and numerical simulation [11,12,13,14,15,16,17,18,19,20,21,22,23] to conduct extensive investigations into the flow characteristics of molten steel in the mold and the mechanisms of protective slag entrainment. Existing studies have shown that slag entrainment behavior in the mold is influenced by a variety of process parameters, including casting speed [8,9,19,20,24,25], nozzle shape [26,27,28,29,30], protective slag properties [24], and nozzle immersion depth [10,11,12]. Among these factors, the submerged entry nozzle, as a component whose parameters can be relatively easily adjusted, has been extensively studied with respect to its shape, angle, immersion depth, and other parameters influencing the flow field and slag entrainment behavior in the mold, and has become a research hotspot in this field. Lu Q. T. et al. [8] investigated the effects of nozzle angle and immersion depth on slag entrainment behavior using a water model. Their results showed that as the nozzle immersion depth and angle decreased, the meniscus velocity and liquid surface fluctuation amplitude increased significantly, leading to a pronounced enhancement of slag entrainment. Srinivas P. S. et al. [10] pointed out that under conditions of greater nozzle immersion depth, the interaction between rising bubbles and eddies can weaken shear effects and eddy structures, thereby reducing the frequency of slag entrainment. Ren Lei et al. [11] employed PIV technology to obtain time-averaged velocity distributions within the mold and verified the flow field characteristics and meniscus fluctuation behavior under different casting speeds. Wu Yingdong et al. [12] further demonstrated experimentally that reducing the nozzle immersion depth significantly increases the frequency of slag entrainment. Bai H. et al. [26] found that, compared with straight nozzles, enlarged nozzles can significantly reduce meniscus velocity and flow intensity, which is beneficial for improving flow field stability within the mold and reducing the risk of slag entrainment. Bai H. T. et al. [27] suggested that the influence of nozzle structure on the flow field is mainly concentrated in the upper region of the mold, while its effect weakens at greater depths. Chen Gang et al. [29] reported that convex nozzles have a greater influence on liquid surface fluctuations than concave nozzles. Xiong Xiao et al. [30] used a water model to study the effects of rectangular, elliptical, and square nozzles on molten steel flow in the mold and found that elliptical nozzles perform better at high casting speeds. Although existing studies have systematically revealed the qualitative effects of submerged entry nozzle structural parameters on the flow field and slag entrainment behavior in the mold, systematic quantitative investigations of key process parameters—such as the critical casting speed and critical immersion depth for slag entrainment under different nozzle structures—are still lacking. This limitation, to some extent, restricts the direct applicability of existing research results to industrial production.

In this study, a wide slab continuous casting mold from a steel plant is taken as the research object. A 1:4-scale water model is established, and a high-speed camera is employed to visualize meniscus fluctuations and slag entrainment processes within the mold. By systematically comparing the slag entrainment behavior of elliptical and circular submerged entry nozzles under different casting speeds and immersion depths, the critical casting speed and critical immersion depth for slag entrainment are determined. An alternating control variable method is used to divide the operating conditions into a “safe zone,” in which slag entrainment does not occur, and a “slag entrainment zone,” in which oil droplets fall. Based on these results, numerical simulations are conducted to further analyze the molten steel flow field structure within the mold. The intrinsic mechanisms underlying the differences in slag entrainment caused by different nozzle shapes are revealed in terms of flow field symmetry, the structure of the upper recirculation zone, and flow characteristics near the meniscus. This research guides nozzle selection and process optimization in wide slab continuous casting and contributes to the improvement of slab surface quality.

## 2. Methods

### 2.1. Establishment of the Mold Water Model

The water model apparatus used in this study is based on the physical model previously established by Ren et al. [31] for investigating multiphase flow behavior in a wide-width continuous casting mold. The same mold geometry and similarity criteria are adopted in the present work.

**Figure 1:**
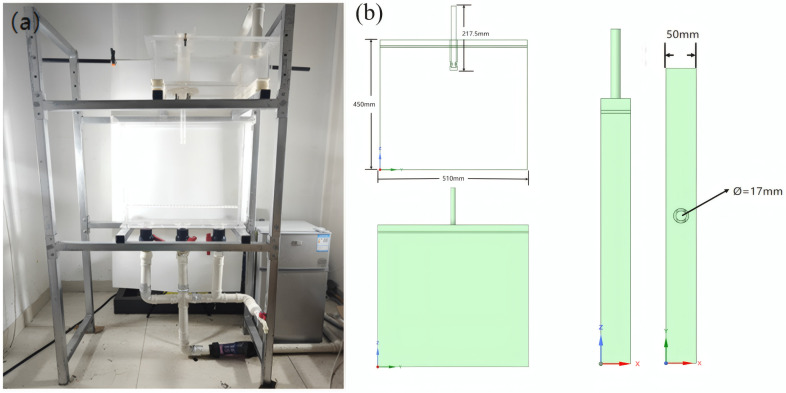
Schematic diagram of the water model structure of the wide slab continuous casting mold, adapted from Ref. [31]. (**a**) Physical view of the experimental setup; (**b**) Geometry of the mold water model.

The water model prototype is a wide slab continuous casting mold for a steel plant, measuring 2040 mm in width and 200 mm in thickness. The water model was designed with a similarity ratio of 1:4, with dimensions of 450 mm in length, 510 mm in width, and 50 mm in thickness, as shown in Figure 1. Table 1 lists the parameters of the prototype and the model.

The physical model was primarily scaled according to Froude number (Fr) similarity to accurately reproduce the dominant inertial and gravitational forces that govern bulk flow patterns and surface fluctuations in the mold. The Reynolds number (Re) in the model was maintained well within the fully turbulent regime (Re > 10^5^), ensuring that viscous effects were negligible and comparable to those in the prototype conditions.

### 2.2. Shape of the Submerged Entry Nozzle and Selection of Experimental Oil

**Figure 2:**
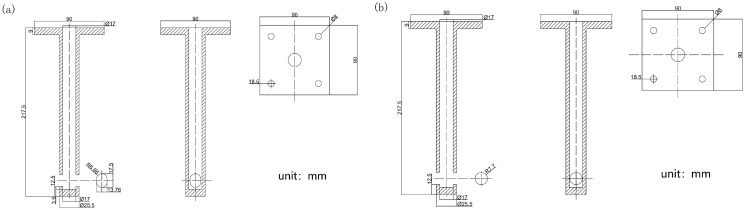
Schematic diagram of the submerged entry nozzle structures (**a**) Elliptical nozzle; (**b**) Circular nozzle, adapted from Ref. [31].

The design of the submerged entry nozzle should consider its compatibility with casting speed and slab dimensions, as well as its stability during long-term operation. Elliptical and circular submerged entry nozzles were selected for comparison in this study, as these two shapes are widely applied in wide slab continuous casting, and their flow field characteristics have a pronounced influence on meniscus fluctuations and slag entrainment behavior. The asymmetric flow field generated by the elliptical submerged entry nozzle tends to cause greater fluctuations in the meniscus. In contrast, the inherent symmetry of the circular nozzle promotes a more uniform flow distribution, thereby stabilizing the meniscus. Figure 2 shows the structural diagrams of the two types of submerged entry nozzles.

To make the two nozzles comparable in flow rate, their outlet areas were designed to be almost the same—about 188.77 mm^2^ for the elliptical nozzle and 186 mm^2^ for the circular one. The 1.5% difference is small enough to have little effect on the experimental results. Any minor deviations would primarily be reflected in the velocity distribution at the outlet and would not significantly impact the overall comparison of flow patterns.

In this experiment, dimethyl silicone oil was used to simulate the mold flux in continuous casting, while water at room temperature represented the molten steel flow in the mold. Silicone oils with different kinematic viscosities correspond to types of mold flux in actual production, allowing the effects of nozzle shape on flow patterns under various casting conditions to be studied, thereby providing a theoretical basis for design optimization. The interactions between molten steel and mold flux, as well as between water and dimethyl silicone oil, are mainly governed by viscous forces. By matching the kinematic viscosity ratio between the two fluids, the water model can reliably replicate the flow behavior observed in actual production. In this study, dimethyl silicone oils with kinematic viscosities of 5 × 10^−6^ m^2^·s^−1^, 50 × 10^−6^ m^2^·s^−1^, and 100 × 10^−6^ m^2^·s^−1^ were employed. The physical properties of water, molten mold flux, and molten steel are summarized in Table 2.

### 2.3. Image Recognition System and Processing Method

To quantitatively investigate meniscus fluctuation and slag entrainment behavior, an image recognition technique was developed and incorporated into the physical water model experiments. In these experiments, silicone oil was employed to simulate the mold flux, forming an oil layer of 5 mm in thickness. Each operating condition was repeated three to five times to ensure experimental reproducibility. The retention behavior of the mold flux was evaluated by monitoring the penetration of oil droplets at the water–oil interface.

**Figure 3:**
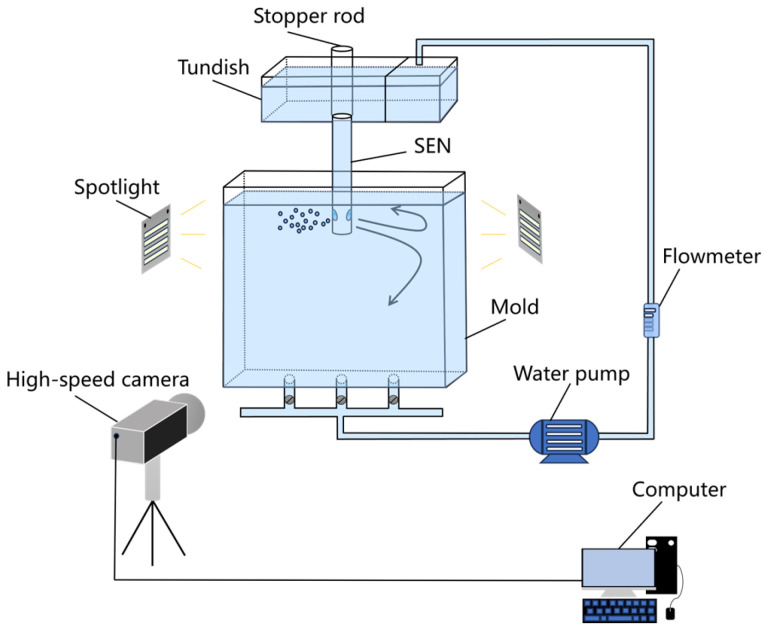
Schematic diagram of the image recognition system experimental setup, adapted from Ref. [31].

**Figure 4:**
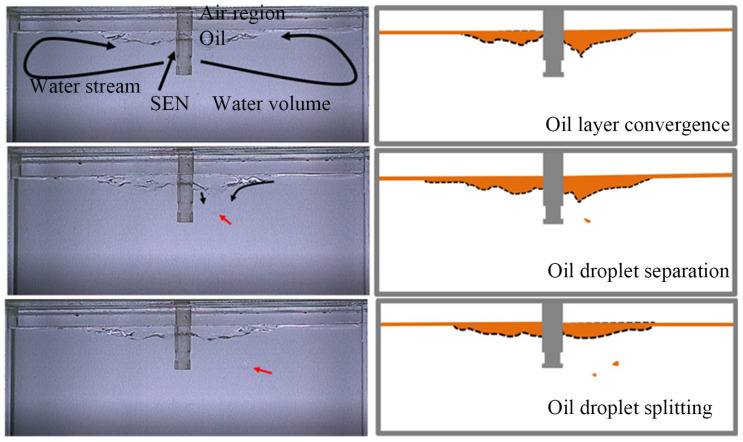
Schematic diagram of the slag entrainment process.

The schematic of the image recognition system is shown in Figure 3. A high-speed camera (VW-900, KEYENCE Corporation, Osaka, Japan) was positioned perpendicular to the wide face of the mold and operated at 1000 frames per second (fps) with a spatial resolution of 0.05 mm·pixel^−1^. The captured videos were processed using an open-source computer vision library (OpenCV, Python 3.9) to automatically extract the instantaneous meniscus profile and identify events of slag droplet entrainment. As shown in Figure 4, under the condition of an elliptical nozzle with an immersion depth of 40 mm, a casting speed of 0.5 m/min, and a silicone oil viscosity of 5 × 10^−6^ m^2^·s^−1^, no droplet detachment was observed, indicating a non-slag-entrainment safe zone. In contrast, when droplets were observed to penetrate the water phase, the condition was defined as a slag entrainment zone.

The time series of meniscus elevation was extracted from the processed image sequence, and the fluctuation intensity was quantified using the root-mean-square (RMS) deviation from the mean liquid level. The frequency of slag droplet entrainment was determined by counting the number of droplets detected entering the water phase within a fixed observation window of 300 s.

## 3. Numerical Simulation

### 3.1. Basic Assumptions

In actual wide slab casting, considering the complexity of on-site conditions, the present study makes the following assumptions to simplify the model and improve computational efficiency:

1. The liquid is assumed to be an isotropic, incompressible Newtonian fluid with constant viscosity, specific heat, and thermal conductivity;
2. The effects of slab shrinkage and mold oscillation on the flow behavior of molten steel are neglected;
3. The mold meniscus is assumed to be flat and adiabatic;
4. The physical properties of molten steel are assumed to be uniform in all directions during the continuous casting process.

These assumptions enable a simplified calculation, allowing for a focus on the influence of nozzle shape on flow behavior. The numerical simulations were carried out using ANSYS Fluent 2022 R1. Under the above assumptions, the pressure–velocity coupling was handled using the SIMPLE algorithm, and the momentum equations were discretized using a second-order upwind scheme.

### 3.2. Governing Equations

Fluid Flow Equations:

(1) $$\frac{\partial \rho }{\partial t}+\;\nabla \;\cdot \;\left(\rho u\right)=0$$

Fluid flow models are primarily based on the continuity equation and the momentum equation:

(2) $$\frac{\partial }{\partial t}\left(\rho u\right)+\;\nabla \;\cdot \;\left(\rho uu\right)=-\nabla p+\;\nabla \;\cdot \;\left[\mu_{eff}\left(\nabla u+\;\nabla u^{T}\right)\right]\;+\;\rho g+\;\rho g\beta_{T}\left(T-\;T_{ref}\right)+\;S_{m}+\;F_{mag}$$

where ρ is density, in kg/m^3^; *t* is the time variable, in s; *u* is the fluid velocity vector, in m/s; *p* is pressure, in Pa; *g* is the gravitational acceleration, in m/s^2^; $\beta_{T}$ is the thermal expansion coefficient, in K^−1^; *T* is temperature, in K; and $T_{\mathrm{ref}}$ is the reference temperature, in K, set to the liquidus temperature; $\rho g\beta_{T}\left(T-\;T_{ref}\right)$ represents the thermal buoyancy force; $S_{m}$ denotes the momentum source term associated with the mushy zone, and $F_{mag}$ represents the magnetic force term induced by an external electromagnetic field (in the present study, solidification and electromagnetic effects were neglected; therefore, $S_{m}$ and $F_{\mathrm{mag}}$ were set to zero).

(3) $$\mu_{eff}=\mu_{l}+\mu_{t}=\mu_{l}+\rho C_{\mu }\frac{k^{2}}{\varepsilon }\;$$

where $\mu_{l}$ and $\mu_{t}$ represent the laminar viscosity and turbulent viscosity, respectively, in kg·m^−1^·s^−1^; $C_{\mu }$ = 0.09 (an empirical constant); *k* is the turbulent kinetic energy, in m^2^·s^−2^; and ε is the turbulent dissipation rate, in m^2^·s^−3^.

Turbulence is controlled by the realizable *k* − ε two-equation model. The equations for *k* and ε are as follows:

(4) $$\frac{\partial }{\partial t}\left(\rho k\right)+\;\nabla \;\cdot \;\left(\rho ku\right)=\;\nabla \;\cdot \;\left[\left(\mu_{l}+\;\frac{\mu_{t}}{\sigma_{k}}\right)\nabla k\right]+\;G_{k}+\;G_{b}-\;\rho \varepsilon +\;S_{k}$$

(5) $$\frac{\partial }{\partial t}\left(\rho \varepsilon \right)+\nabla \;\cdot \;\left(\rho \varepsilon u\right)=\nabla \;\cdot \;\left[\left(\mu_{l}+\frac{\mu_{t}}{\sigma_{\varepsilon }}\right)\nabla \varepsilon \right]+C_{1\varepsilon }\frac{\varepsilon }{k}\left(G_{k}+C_{3\varepsilon }G_{b}\right)-C_{2\varepsilon }\rho \frac{\varepsilon^{2}}{k}+S_{\varepsilon }$$

Here, $G_{k}$ is the turbulent kinetic energy generated by the mean velocity gradient; $G_{b}$ is the turbulent kinetic energy generated by buoyancy; $C_{1\varepsilon }$, $C_{2\varepsilon }$ and $C_{3\varepsilon }$ are empirical constants with recommended values of 1.44, 1.92, and −0.33, respectively; $\sigma_{k}$ and $\sigma_{\varepsilon }$ are the turbulent Prandtl numbers for *k* and ε, respectively, with recommended values of 1.0 and 1.3; $S_{k}$ and $S_{\varepsilon }$ are source terms.

### 3.3. Boundary Conditions

(1) The inlet is defined as a velocity inlet. (2) The outlet of the computational domain is defined as a pressure outlet. Based on the principle of mass flow conservation, the relationship between the flow velocity of molten steel at the inlet and the casting speed is given by the following conversion formula:

(6) $$\nu_{\mathrm{inlet}}=\;\frac{\nu_{\mathrm{outlet}}s_{\mathrm{outlet}}}{s_{\mathrm{inlet}}}$$

In the equation, $\nu_{inlet}$ represents the velocity at the mold inlet (in m/s); $\nu_{\mathrm{outlet}}$ is the casting speed (in m/min), and $s_{\mathrm{inlet}}$ and $s_{\mathrm{outlet}}$ are the inlet and outlet areas of the mold, respectively. (3) The mold liquid level is set to a free surface, with zero shear force applied. (4) Both the mold wall and nozzle wall are treated as no-slip walls, with the standard wall function used near the wall. (5) Transient simulations were performed with a fixed time step of Δt = 0.01 s. Each case was simulated for a total physical time of 20 s to ensure that a statistically steady state was reached.

### 3.4. Grid Independence

**Figure 5:**
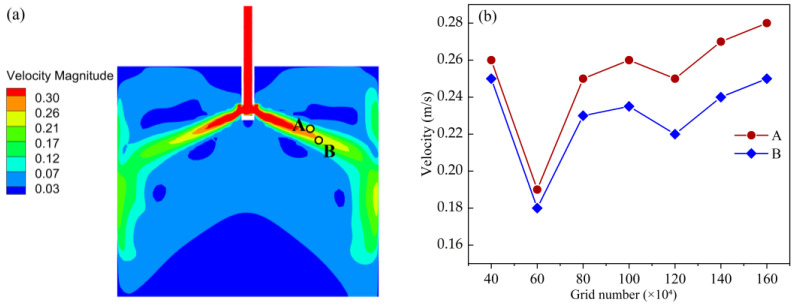
Grid independence verification. (**a**) Locations of points A and B; (**b**) Verification results.

To ensure that the numerical simulation results are independent of the grid size and time step, and to improve the overall computational accuracy, both grid and temporal independence tests were performed. The computational domain was discretized using a structured hexahedral mesh, with local refinement applied in the region near the nozzle outlet and the free surface. In this study, particular attention was given to analyzing the distribution of the flow field within the mold. A cross-section parallel to the yoz-plane at x = 0.025 m was selected, where two monitoring points, A and B, were defined, as shown in Figure 5a.

Under identical operating conditions, the total number of grids was gradually increased from 4.0 × 10^5^ to 1.6 × 10^6^, and the corresponding velocity values at these two points were compared to assess grid independence. As illustrated in Figure 5b, the calculated velocities at both points become nearly constant when the grid number exceeds approximately 1.2 × 10^6^, with variations less than 3%. Therefore, a grid system containing about 1.4 × 10^6^ elements was adopted for subsequent simulations to ensure a good balance between computational accuracy and efficiency.

### 3.5. Model Validation

**Figure 6:**
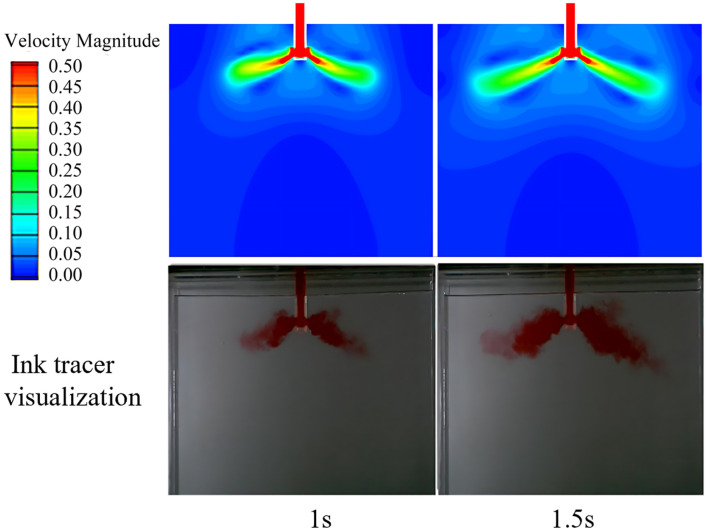
Transient flow inside the mold at different moments.

Through transient analysis, the differences between physical simulation and numerical simulation under the same working conditions were compared and verified. At the same time, the rationality of the boundary conditions set in the numerical simulation was confirmed. Taking a casting speed of 0.6 m/min and an immersion depth of 45 mm as an example, Figure 6 presents the transient flow within the mold at various time intervals. During the 0–1 s period, the molten steel flows from the nozzle inlet through the submerged entry nozzle to one-quarter of the mold’s width; during the 1.0–1.5 s period, the jet further expands towards the narrow side. Overall, the evolution process of fluid flow in the numerical simulation is consistent with the results of the ink tracer test in the water model, thereby verifying the accuracy of the simulation boundary conditions. It is worth noting that the numerical simulation did not account for the buoyancy effect or intermolecular interactions, which led to some discrepancies between the observed tracer diffusion patterns and the simulation results. However, these differences have a relatively minor impact on the overall flow characteristics.

## 4. Results and Discussion

### 4.1. Water Model Experimental Results and Discussion

#### 4.1.1. Comparison of Flow Patterns During Initial Casting with Elliptical and Circular Nozzles

**Figure 7:**
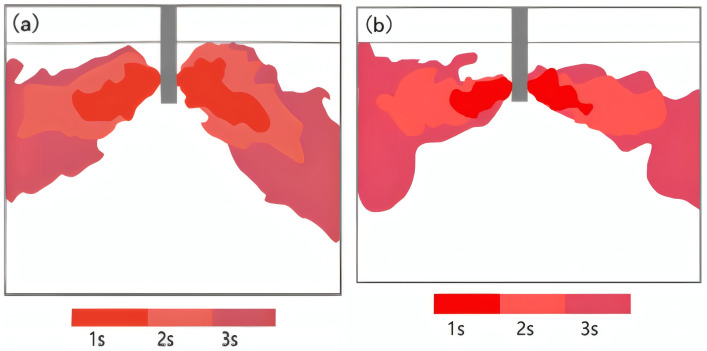
Instantaneous flow patterns for different nozzles. (**a**) Elliptical nozzle; (**b**) Circular nozzle.

To investigate the transient flow patterns in the mold, a red ink tracer was injected into the tundish. The tracer entered the mold through the submerged entry nozzle (SEN), which connects the bottom of the tundish to the mold. Figure 7 shows the instantaneous flow fields for elliptical and circular nozzles under a casting speed of 0.45 m/min and a nozzle immersion depth of 40 mm. Under these working conditions, both types of nozzles exhibit good symmetry in the flow field. The water flow takes about 2.5 s to reach the narrow side of the mold from the elliptical nozzle. In contrast, the circular nozzle, due to its more concentrated outflow velocity, has a relatively shorter transmission time, taking approximately 2.2 s.

#### 4.1.2. Critical Slag-Entrainment-Free Immersion Depth

**Figure 8:**
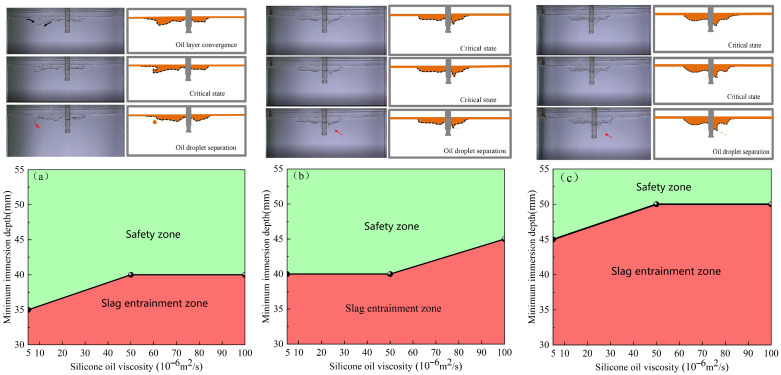
Critical immersion depth of the elliptical nozzle to prevent slag entrainment at different casting speeds. (**a**) 0.50 m/min; (**b**) 0.55 m/min; (**c**) 0.60 m/min.

Taking the immersion depth and the viscosity of silicone oil as variables, Figure 8 shows the critical immersion depth for no slag entrainment of the elliptical nozzle at different casting speeds. As shown in the figure, the region where slag entrainment occurs is defined as the slag entrainment zone, whereas the region without slag entrainment is defined as the safe zone. As the casting speed increases, the area of the safety zone gradually decreases, indicating an increased risk of slag entrainment. Furthermore, when the casting speed is 0.5 m/min, a safe range of nozzle immersion depths exist that correspond to different viscosities of silicone oil. This analysis result provides clear operational guidance for setting the immersion depth of the nozzle in actual production. For example, under operating conditions with a casting speed of 0.5 m/min and a silicone oil viscosity of 50 × 10^−6^ m^2^·s^−1^, if the immersion depth of the nozzle is less than 40 mm, slag entrainment will occur. Conversely, when the immersion depth exceeds 40 mm, slag entrainment can be effectively avoided.

**Figure 9:**
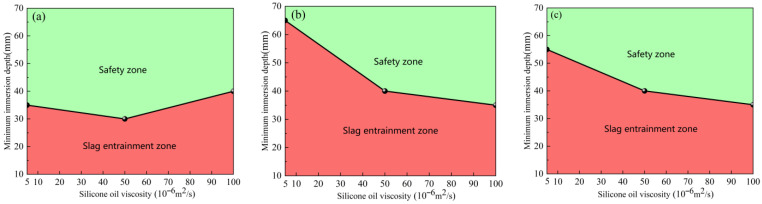
Critical immersion depth of the circular nozzle to prevent slag entrainment at different casting speeds. (**a**) 0.50 m/min; (**b**) 0.55 m/min; (**c**) 0.60 m/min.

The critical immersion depth of the circular nozzle, without slag entrainment, is illustrated in Figure 9, where immersion depth and viscosity of silicone oil are the variables. When the casting speed exceeds 0.55 m/min, the phenomenon of oil droplets continuously detaching from the oil layer and remaining in the molten steel occurs at the circular nozzle, which seriously affects the quality of the cast slab in actual production. Therefore, in the water model experiment, the casting speed of the circular nozzle should be controlled below 0.55 m/min to avoid such problems. When the casting speed is 0.5 m/min, the critical immersion depth varies slightly under different viscosities. After increasing the casting speed, the variation becomes significant. As the casting speed increases, the safe zone area decreases, the probability of oil droplets falling increases, and the likelihood of slagging in actual production also increases.

#### 4.1.3. Critical Slag-Entrainment-Free Casting Speed

**Figure 10:**
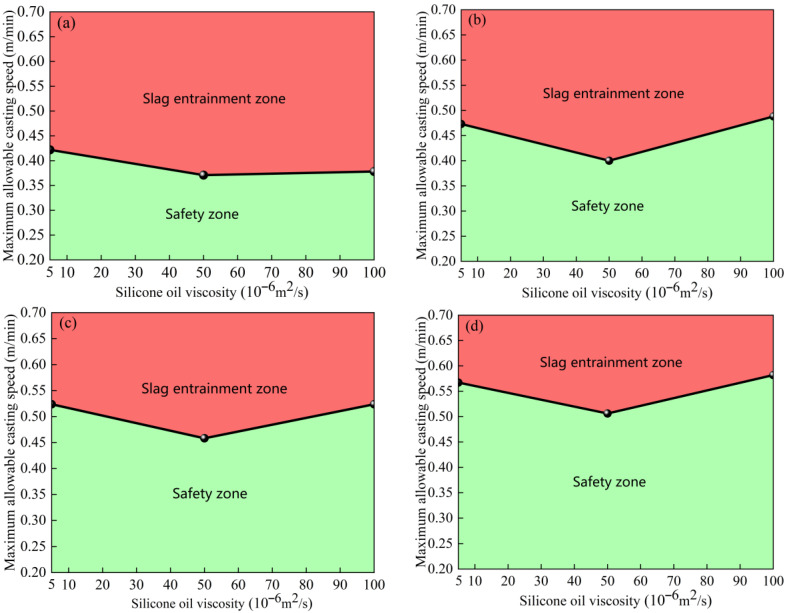
Critical casting speed for the elliptical nozzle at different immersion depths. (**a**) 30 mm; (**b**) 35 mm; (**c**) 40 mm; (**d**) 45 mm.

**Figure 11:**
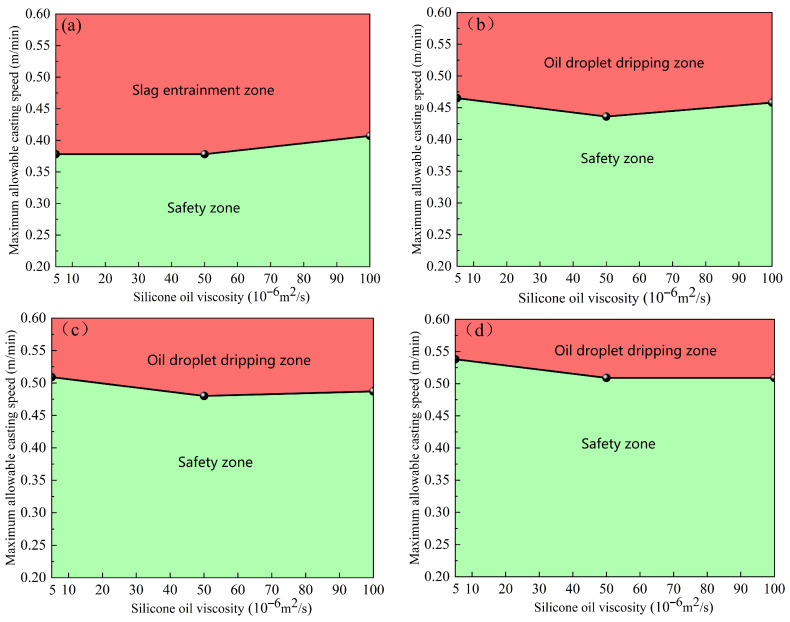
Critical casting speed for circular nozzle at different immersion depths. (**a**) 30 mm; (**b**) 35 mm; (**c**) 40 mm; (**d**) 45 mm.

By comparing the critical casting speeds of the elliptical and circular nozzles under different working conditions, the oil drop falling zone and the safe zone can be identified. As the immersion depth of the nozzle increases, the area of the safety zone gradually expands, and the risk of slag entrainment decreases. Figure 10 shows the critical casting speeds of the elliptical nozzle at various immersion depths. As shown in Figure 10a, when the immersion depth of the nozzle is 30 mm, the critical casting speed range without slag entrainment can be obtained under different silicone oil viscosities, which is of guiding significance for setting the casting speed in actual production. Figure 11 illustrates the critical casting speed of the circular nozzle at various immersion depths. Under the same process parameters, the critical casting speed of the circular nozzle increases to 0.378 m/min before the slag entrainment occurs, indicating that the probability of slag entrainment is reduced.

#### 4.1.4. Comparison of Meniscus Fluctuation Between Elliptical and Circular Nozzles

**Figure 12:**
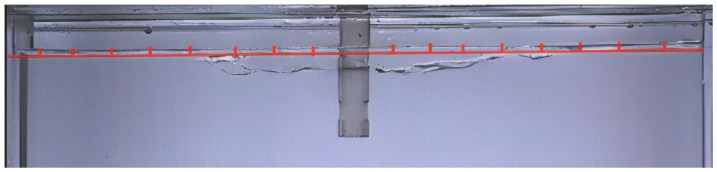
Schematic diagram of meniscus fluctuation measurement.

The fluctuation of the liquid level is a crucial indicator for assessing the activity of the liquid in the mold. If the fluctuation is too small, the liquid level will not be active enough, which is not conducive to the melting of the protective slag and is prone to the formation of a slag ring. At the same time, if the fluctuation is too large, it is easy to cause deep vibration marks on the cast slab and draw the protective slag into the molten steel [32]. Therefore, by analyzing the fluctuation of the liquid surface, the occurrence mechanism of the liquid surface slag entrainment behavior can be explained to a certain extent. Figure 12 illustrates the instantaneous meniscus profile of an elliptical nozzle under conditions of silicone oil viscosity of 5 × 10^−6^ m^2^·s^−1^, an immersion depth of 40 mm, and a casting speed of 0.5 m/min. The solid line represents the working liquid level, that is, the distance from the liquid surface to the upper edge of the mold. Eight monitoring points are evenly set on both sides of the mold. The amplitude of liquid level fluctuation is defined as the distance that the instantaneous liquid level at each monitoring point deviates from the working liquid level (red line). The average fluctuation value refers to the mean of the instantaneous fluctuation values at each monitoring point within a specific time period, which characterizes the overall fluctuation characteristics of the liquid surface. In this experiment, 20 transient data points within 12.5 s were selected for the analysis of the liquid surface fluctuation. The free surface was recorded using a high-speed camera at a frame rate of 1000 frames per second, and the liquid level contours were extracted through image processing. The camera was calibrated using a ruler to convert pixels into millimeters, allowing for the measurement of instantaneous liquid levels.

In this section, a silicone oil with a kinematic viscosity of 5 × 10^−6^ m^2^·s^−1^ was used as the working fluid to investigate the effect of nozzle geometry on meniscus fluctuations, rather than the influence of fluid viscosity. To avoid interference from viscosity variations, the viscosity of the silicone oil was maintained at a constant level, ensuring the comparability of liquid surface fluctuations under different nozzle shapes. As the effects of varying viscosities on slag entrainment have been systematically analyzed in previous sections, the selected typical viscosity is representative of actual operating conditions.

**Figure 13:**
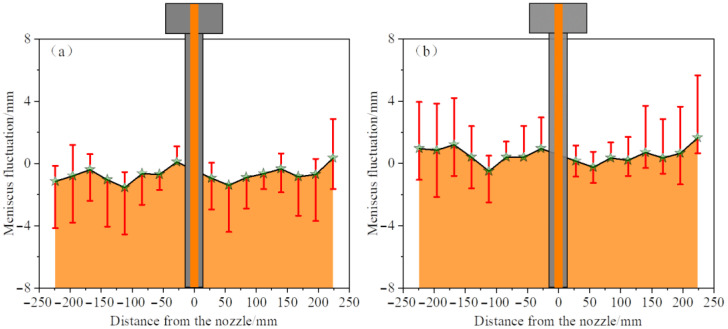
Average meniscus profile and fluctuation of the elliptical nozzle at different casting speeds. (**a**) 0.50 m/min; (**b**) 0.55 m/min.

**Figure 14:**
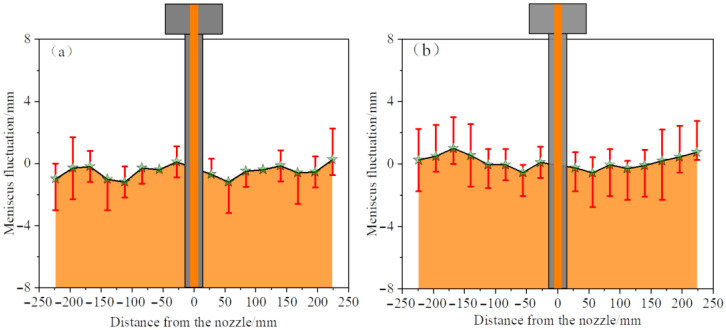
Average meniscus profile and fluctuation of circular nozzle at different casting speeds. (**a**) 0.50 m/min; (**b**) 0.55 m/min.

Figure 13 and Figure 14 compare the average liquid surface profiles of the two types of nozzles at different casting speeds (immersion depth of 40 mm, constant viscosity of silicone oil). When the casting speed of the elliptical nozzle is increased to 0.55 m/min, the average value of the liquid level fluctuation rises significantly, and the overall fluctuation intensity intensifies. The amplitude and range of the fluctuation on the narrow side on the right are particularly significant, indicating that the high casting speed intensifies the local disturbance of the elliptical nozzle. In contrast, the circular nozzle shows a lower and more uniform surface fluctuation amplitude at 0.5 m/min. When the casting speed increases to 0.55 m/min, the impact intensifies due to the upper reflux approaching the oil layer; however, the fluctuation intensity remains lower than that of the elliptical nozzle. Under the same casting speed conditions, the liquid level fluctuation of the elliptical nozzle is consistently greater than that of the circular nozzle, thereby increasing the risk of slag entrainment.

To quantify the intensity of meniscus fluctuation at each monitoring point i within the mold, the Root-Mean-Square (RMS) of the meniscus fluctuation is introduced. Its calculation formula is as follows:

(7) $$\Delta l_{\mathrm{RMS}}=\sqrt{\frac{\sum_{i=1}^{n}{\left(\Delta l_{i}\right)}^{2}}{n}}.$$

The meniscus fluctuation value at monitoring point *i* at each moment is its deviation from the average liquid level, i.e., the instantaneous value of the meniscus profile at monitoring point *i* interpolated with its average value, which is:

(8) $$\Delta l_{i}\;=\;l_{i}\;-{\overset{‾}{l}}_{i}.$$

where $l_{i}$ is the instantaneous value of the meniscus profile at monitoring point *i*, ${\overset{‾}{l}}_{i}$ is the average value of the meniscus profile at monitoring point *i*, and *n* is the number of monitoring times. $\Delta l_{i}$ is the meniscus profile fluctuation value at monitoring point *i*.

**Figure 15:**
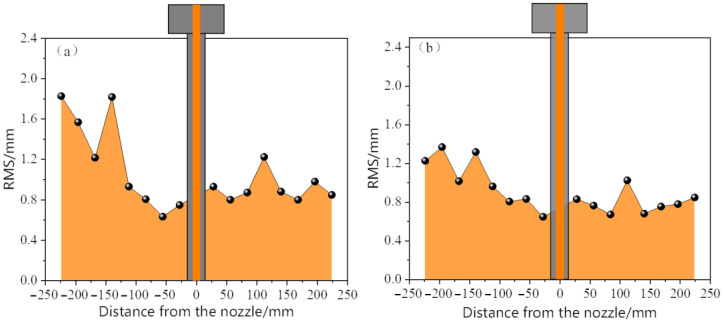
Root mean square (RMS) of meniscus fluctuation in the mold during monitoring time for the elliptical nozzle. (**a**) Immersion depth 30 mm; (**b**) Immersion depth 35 mm.

**Figure 16:**
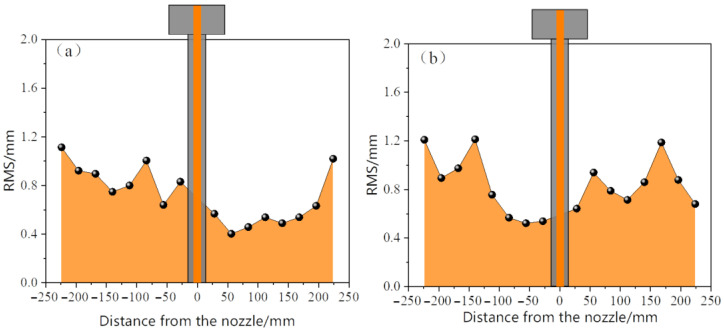
Root mean square (RMS) of meniscus fluctuation in the mold during monitoring time for circular nozzle. (**a**) Immersion depth 30 mm; (**b**) Immersion depth 35 mm.

Figure 15 and Figure 16 compare the distribution of the root mean square (RMS) values of the liquid surface fluctuations of the two types of nozzles at different immersion depths. When the immersion depth was 30 mm, the elliptical nozzle showed significant volatility, with the RMS value of the local monitoring points exceeding 1.6 mm, and the fluctuation non-uniformity was prominent. When the immersion depth increased to 35 mm, the RMS value generally decreased to below 1.2 mm, indicating that the stability of the liquid surface significantly improved with the increase in the immersion depth. In contrast, the RMS values of the circular water inlet remain stable within the range of 0.6 to 1.2 mm at both immersion depths, with a relatively low fluctuation and uniform distribution. An increase in the immersion depth only causes a slight decrease in the fluctuation value. This indicates that the circular nozzle has a lower sensitivity to changes in immersion depth and possesses superior flow field stability. It can be seen from this that the elliptical nozzle is prone to causing stronger liquid surface fluctuations at a shallow immersion depth (30 mm) and requires optimization and control by increasing the immersion depth. In contrast, the circular nozzle exhibits a lower level of liquid surface fluctuations across different immersion depths and features more stable flow characteristics and a lower risk of slag entrainment.

#### 4.1.5. Slag Entrainment Frequency for Different Nozzle Shapes

**Figure 17:**
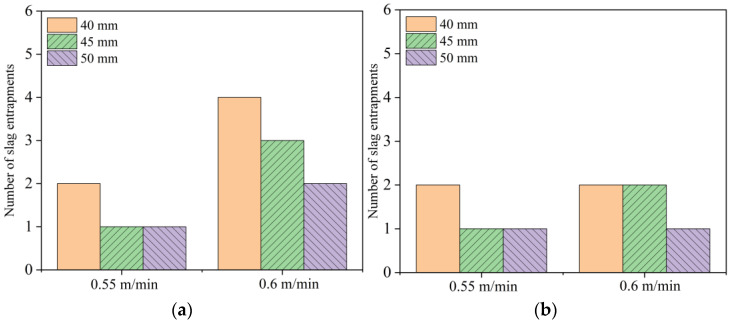
Slag entrainment frequency for different nozzles. (**a**) Elliptical nozzle; (**b**) Circular nozzle.

This study quantitatively analyzed the variation in slag entrainment frequency within 300 s under different working conditions for elliptical and circular nozzles. The results indicate that immersion depth significantly influences slag entrainment behavior, with the frequency of entrainment decreasing as the immersion depth increases. The variation in casting speed also has a significant impact on the risk of slag entrainment. When the casting speed increases from 0.55 m/min to 0.6 m/min, the fluid flow within the mold intensifies, and the overall frequency of slag entrainment rises (as shown in Figure 17). Under the conditions of immersion depths of 40 mm and 50 mm, increasing the casting speed to 0.6 m/min resulted in additional slag entrainment within 300 s, clearly demonstrating the trend that the risk of slag entrainment increases with the rise in casting speed. Under the same process conditions, when the casting speed is 0.55 m/min, the number of slag entrainment times of the elliptical and circular nozzles is similar, with no significant difference; however, when the casting speed increases to 0.6 m/min, the number of slag entrainment times of the elliptical nozzle is significantly higher than that of the circular nozzle.

### 4.2. Numerical Simulation Results and Discussion

#### 4.2.1. Flow Characteristics at the Wide Face Under Different Nozzle Shapes

**Figure 18:**
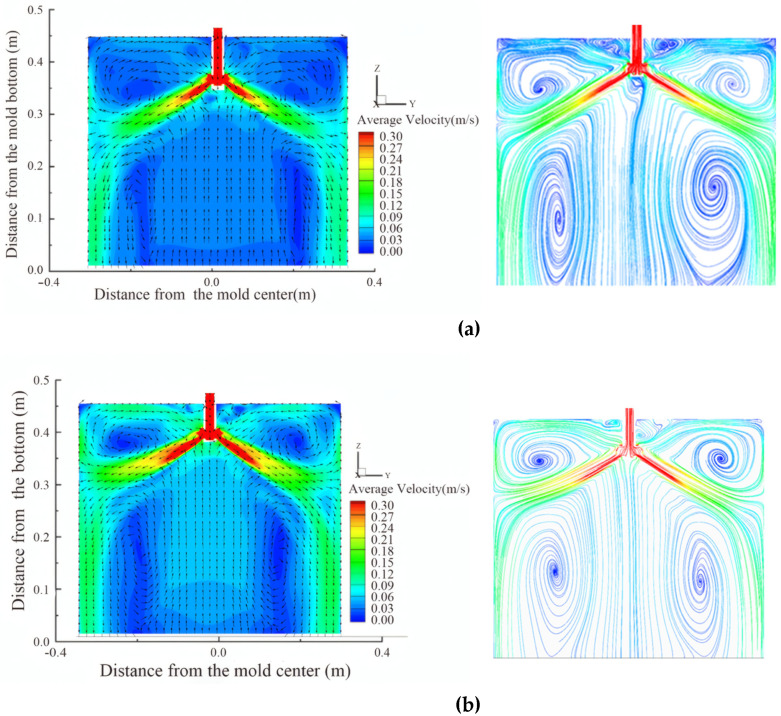
Velocity contour and streamline diagrams for different nozzle shapes. (**a**) Elliptical nozzle; (**b**) Circular nozzle.

Figure 18 illustrates the flow field distribution within the mold under the conditions of a casting speed of 0.5 m/min and an immersion depth of 50 mm, comparing flow fields of elliptical and circular nozzles. It can be observed that the elliptical nozzle has a larger jet angle, resulting in a broader impact range and a more dispersed momentum. The liquid flow forms a symmetrical dual circulation flow field on both sides of the wide face, with the upper circulation being particularly pronounced. The maximum flow velocity near the nozzle is approximately 0.27–0.30 m/s, indicating relatively active surface flow, which is conducive to the melting and lubrication of the protective slag; however, the lower circulation region is relatively shallow.

In contrast, for the circular nozzle, the jet is more concentrated with a smaller angle, and the impact point is slightly lower, forming a deeper and more stable lower circulation flow field. The overall streamlined structure is relatively stable, exhibiting better symmetry. The surface flow velocity is relatively low, which reduces the risk of slag entrainment, although the heat exchange at the liquid surface may be slightly diminished. Overall, the elliptical nozzle promotes enhanced surface flow and the development of the upper circulation, whereas the circular nozzle helps establish a deeper and more stable internal flow structure.

#### 4.2.2. Flow Characteristics at the Narrow Face Under Different Nozzle Shapes

**Figure 19:**
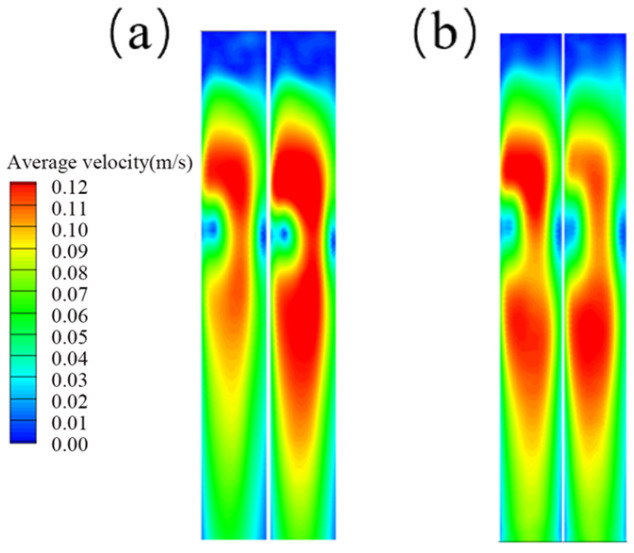
Velocity contours at the left and right narrow faces. (**a**) Elliptical nozzle; (**b**) Circular nozzle.

To investigate the flow patterns within the mold under different nozzle shapes, the nozzle geometry was varied while keeping other operating conditions constant. Figure 19 shows the velocity contour distributions at the left and right narrow faces of the mold for the two nozzle shapes. It can be seen that, under the same operating conditions, the circular nozzle exhibits good symmetry in the velocity distribution on both narrow faces.

**Figure 20:**
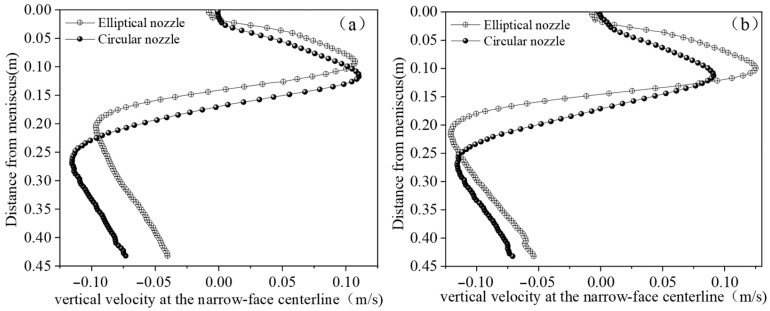
Vertical velocity at the narrow-face centerline under different nozzle shapes. (**a**) Left narrow face; (**b**) Right narrow face.

At the mid-thickness centerlines of the left and right narrow faces (x = 0.025 m), 200 sampling points were extracted along the positive z-axis. The corresponding vertical velocity distributions are presented in Figure 20. In the figure, positive velocity values denote upward flow toward the meniscus, whereas negative values indicate downward flow toward the mold bottom. The location where the velocity changes sign represents the jet impingement point on the narrow face.

As shown, the jet from the elliptical nozzle impinges at a higher position on the narrow face and induces a stronger downward flow, as evidenced by the larger negative velocity peak. This flow pattern redistributes more kinetic energy into the lower recirculation zone, thereby weakening the upward flow toward the meniscus. In contrast, the circular nozzle generates a deeper but more symmetric flow structure, leading to a more balanced momentum distribution between the upper and lower recirculation zones and consequently weaker surface flow intensity.

#### 4.2.3. Liquid Surface Velocity for Different Nozzle Shapes at the Same Casting Speed

**Figure 21:**
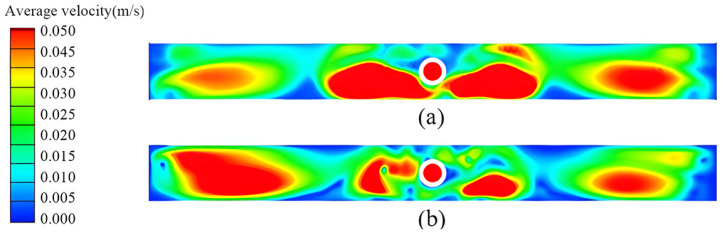
Velocity contours at the free liquid surface. (**a**) Elliptical nozzle; (**b**) Circular nozzle.

Figure 21 presents the velocity contours of the free liquid surface for the two types of nozzles. It can be seen that the elliptical nozzle exhibits higher velocities near the nozzle and poorer symmetry, with the jet striking the upper region of the narrow face after exiting the nozzle, which may easily lead to slag entrainment. In contrast, the circular nozzle shows better symmetry on both sides at the free liquid surface.

**Figure 22:**
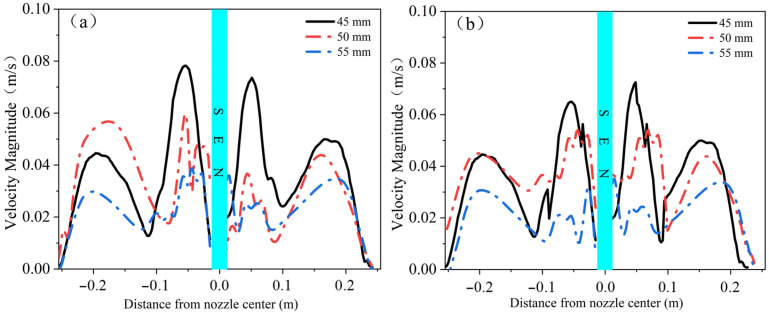
Free surface resultant velocity at different immersion depths. (**a**) Elliptical nozzle; (**b**) Circular nozzle.

As shown in Figure 22, comparing the velocity magnitudes of the free liquid surface for the two types of nozzles reveals that the immersion depth has a significant influence on the flow state of the liquid surface. When the immersion depth is 45 mm, the liquid surface convergence speed of both types of nozzles reaches its maximum, and the risk of slag entrainment is the highest. However, when the immersion depth increases to 55 mm, the liquid surface convergence speed significantly decreases, and the symmetry of the speed distribution is optimal, which helps to reduce the probability of slag entrainment. The velocity distribution of the liquid surface at the elliptical nozzle exhibits apparent asymmetry. It changes sharply with increasing immersion depth, which is prone to cause liquid surface fluctuations, slag entrainment, and other unstable phenomena. In contrast, the velocity distribution of the liquid surface at the circular nozzle is more symmetrical and gentle, which can more effectively maintain the stability of the liquid surface and thereby reduce the probability of slag entrainment.

#### 4.2.4. Comparison of Vortex Core Positions and Slag Entrainment Risk Under Different Nozzle Shapes

Numerous scholars at home and abroad have conducted numerical simulation studies on slag entrainment in the mold; however, there are relatively few studies on the variation in the vortex center position. Given the apparent shortcomings of the vortex identification method based on vorticity extraction, new identification methods have emerged, such as the Q criterion and the Ω criterion, among others, which are based on the evolution of the eigenvalues of the velocity gradient [33,34,35,36]. This study innovatively employs the streamline tracing method to extract the coordinates of vortex centers, with a spatial resolution reaching sub-grid scale (less than 0.5 times the grid size), enabling precise capture of micro-scale vortex center positions.

**Figure 23:**
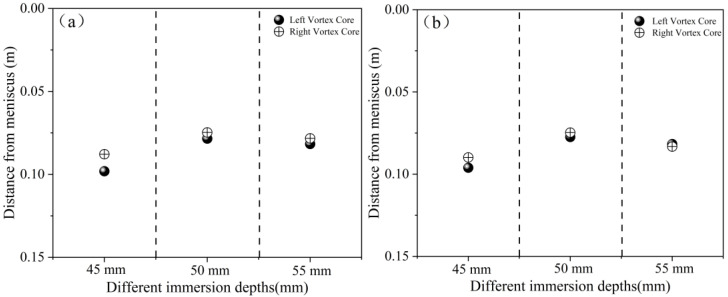
Distance of vortex core from meniscus. (**a**) Elliptical nozzle; (**b**) Circular nozzle.

Figure 23 compares the distance characteristics from the vortex center of the two types of nozzles to the meniscus. When the immersion depth is 50 mm, the vortex core of the elliptical nozzle is closest to the meniscus (left/right: 0.077 m/0.075 m), while when the immersion depth is 45 mm, the vortex core of the circular nozzle is farthest from the meniscus (left/right: 0.098 m/0.088 m). As the immersion depth increases, the vortex centers of both types of nozzles move downward, which helps suppress the upward movement of bubbles, enhances the stability of the liquid surface, and reduces the risk of slag entrainment. Under the same immersion depth, the circular nozzle has better symmetry of the left and right vortex centers (with a deviation of less than 5%) than the elliptical nozzle, indicating that the deep-seated reason why the nozzle shape affects the occurrence of slag entrainment is that it influences the symmetry of the upper recirculation flow within the mold downstream of the nozzle outlet, thereby affecting the flow stability near the meniscus.

## 5. Conclusions

This study systematically investigated the effects of elliptical and circular submerged nozzles on the entrapment behavior of protective slag in a wide slab mold using a 1:4-scale water–oil physical model combined with numerical simulation. The main conclusions are as follows:

1. Using an alternating control variable method, the “safe zone” without entrapment and the “entrapment zone” where oil droplets occur were quantitatively defined. Under the given nozzle immersion depth and protective slag viscosity, the maximum allowable casting speed range can be determined, providing a basis for industrial production parameter control.
2. The root mean square (RMS) of the liquid surface fluctuation effectively characterizes the activity level of the meniscus flow. Its value decreases with increasing immersion depth and increases with increasing casting speed, showing a good correlation with the frequency of entrapment.
3. Compared with elliptical nozzles, circular nozzles form a more symmetrical flow field structure in the upper recirculation zone (left-right vortex center deviation less than 5%), resulting in higher flow field stability near the meniscus, thus creating flow conditions unfavorable to entrapment and exhibiting a lower entrapment frequency.
4. Based on the combined experimental and numerical analysis results, it is recommended to prioritize the use of circular nozzles in wide slab continuous casting, and to combine them with a larger immersion depth (>40 mm) and a moderate casting speed (<0.55 m/min) to improve the stability of the flow field in the mold and the surface quality of the slab.

## Figures and Tables

**Table 1:** Parameters of the mold prototype and water model, adapted from Ref. [31].

Parameter	Mold Prototype	1/4-Scale Water Model
Mold Cross-section	2040 mm × 200 mm	510 mm × 50 mm
Nozzle Inner Diameter	70.0 mm	17.5 mm
Fluid Density	6932 kg·m^−3^ (1490 °C)	998 kg·m^−3^ (20 °C)
Fluid Viscosity	5.23 × 10^−3^ Pa·s (1490 °C)	1.00 × 10^−3^ Pa·s (20 °C)

**Table 2:** Physical Properties of the Liquid Phase, adapted from Ref. [31].

Liquid Phase	ρ (kg·m^−3^)	μ (10^−3^ Pa·s)	υ (10^−6^ m^2^·s^−1^)
Water (20 °C)	998	1.00	1.00
Liquid Slag (1490 °C)	2890	110.00	38.06
Liquid Steel (1490 °C)	6932	5.23	0.75

## Data Availability

The original contributions presented in this study are included in the article. Further inquiries can be directed to the corresponding author.
